# How much does lumbar fusion change sagittal pelvic tilt in individuals receiving total hip arthroplasty?

**DOI:** 10.1186/s42836-019-0014-4

**Published:** 2019-11-29

**Authors:** Gangyong Huang, Guanglei Zhao, Kangming Chen, Yibing Wei, Siqun Wang, Jun Xia

**Affiliations:** Department of Orthopaedic Surgery, Huashan Hospital, Fudan University, 12th Wulumuqi Middle Road, Jing’an District, Shanghai, China

**Keywords:** Total hip arthroplasty, Spinal fusion, Sagittal pelvic tilt

## Abstract

**Background:**

This study primarily aims to examine the effect of lumbar fusion on changes in sagittal pelvic tilt (SPT) in total hip arthroplasty (THA) patients.

**Methods:**

We reviewed 19 hip osteoarthritic patients undergoing THA with or without lumbar fusion. The gender, age, primary disease, Deyo comorbidity score, and year of surgery were sorted and matched. All patients were followed up for at least 12 months. They were compared in terms of the SPT angle, Harris hip score (HHS) and complications.

**Results:**

On average, the patients receiving lumbar fusion had a − 3.9 (95% CI − 7.7 to − 1.5) degrees of SPT before THA and − 2.7 (95% CI − 6.5 to 1.1) degrees postoperatively, and the THA patients without lumbar fusion averaged 2.5 (95% CI − 0.1 to 5.0) degrees and 4.2 (95% CI 2.0 to 6.4) degrees, respectively. In the lumbar fusion patients, the mean SPT was − 3.9 (95% CI − 9.9 to 2.0) degrees with L5S1 fusion and − 4.0(95% CI − 10.0 to 2.1) degrees without L5S1 fusion on the standing radiograph before THA (t = 0.01, *P* = 0.99). The mean SPT was − 1.2 (95% CI − 4.9 to 2.6) degrees with one- and two-segment fusion and − 10.0 (95% CI − 18.5 to 1.5) degrees with three- and four-segment fusion before THA (t = 2.60, *P* = 0.02). There was no statistically significant difference in cup inclination and cup anteversion after THA between the lumbar fusion and control groups. These patients in the two groups achieved a similar HHS 12 months after THA despite the fact that they had different SPT and HHS before THA.

**Conclusion:**

Lumbar fusion appears to increase the posterior SPT by approximately 6 degrees in the patients undergoing THA. Lumbar fusion of more than two segments is a predictor of more posterior SPT changes, but fusion of L5S1 is not.

## Introduction

Disorders of the adult hip and spine are common and the demand for both total hip arthroplasty (THA) and spinal arthrodesis is projected to increase in the future in China. It has been reported that 2 to 5% of patients receiving spinal arthrodesis also underwent THA [1, 2]. Recent studies showed that post-THA complications, especially dislocations, tended to be on the rise in patients with spinal deformities and rigid sacropelvis [1, 3, 4]. It is well-known that the anteversion and inclination of the cup are critical in THA for guaranteeing free movement without impingement and preventing dislocations. To quantitatively assess the cup anteversion and inclination, the anatomic landmarks of the pelvis are commonly used. We must note that these landmarks functionally change as the sagittal pelvic tilt (SPT) varies [5–7]. SPT is the pelvic tilt quantitatively based on the anterior pelvic plane angle, which is the angle between the vertical line and the line from the pubic symphysis to the center of the anterior superior iliac spines. Aging, spinal deformity and rigid sacropelvis can change the SPT and upset the sagittal balance of pelvis [8, 9]. Moreover, various positions, such as lying, sitting and standing, may affect the sagittal balance of the pelvis [10]. In 90% of cases, the posterior SPT with corresponding hyperextension is associated with a less-than-10-degree change in the hip range of motion after surgery. However, if a change in pelvic tilt is more than 20 degrees, surgeons need to adjust the orientation of the implant to avoid the risk of edge-loading and impingement when a conventional prosthesis is used [11]. In extreme cases, dislocation is possible with THA due to excessive pelvic tilt [12].

When the spinal movement is normal, SPT will adapt to the change in lumbar lordosis to minimize the range of motion of the hip on the sagittal plane [7]. Patients with spinal fusion had low flexibility at the lumbosacral junction. A long spinal fusion or the inclusion of the pelvis in fusion critically impacts the hip-spine biomechanics and significantly affects the compensatory ability in the stand-to-sit transition [4, 13]. Several studies examined the relationship between lumbar spine deformities and functional pelvic orientation in individuals with THA [4, 14, 15], yet limited comparative data have been available on the specific impact of primary THAs on the functional recovery of the hip and exact change (degrees) in SPT.

This study was designed to measure the effect of lumbar fusion on SPT before and after THA and to further evaluate the impact of THA on the functional recovery of the hip.

## Methods

Enrolled in the study were 19 patients (including 19 hips) with hip osteoarthritis (involving 19 hips) who had undergone primary THA from Jan 2007 to July 2016, and had a history of lumbar fusion surgery. The study was approved by the institutional review board of the hospital. All patients met the inclusion criteria: (1) older than 30 years, (2) diagnosed with hip osteoarthritis, (3) had a leg discrepancy of less than 2 cm, (4) had prior lumbar fusion surgeries. Exclusion criteria included: (1) had a metal implant in the contralateral hip, (2) the last follow-up was less than 12 months after surgery, and (3) had an indefinite lumbosacral fusion and was in the fusion group. We matched the 19 patients with prior fusion one-to-one to the 19 control patients who had undergone primary THA with no history of spinal fusion. The two groups were grossly matched in terms of age (±5 years), gender, primary disease, Deyo comorbidity score (±0.1) [16], surgeries and year of surgery.

The mean age of patients in fusion group at the time of THA was 60.8 ± 7.3 years, and 9 patients were female. The mean body mass index (BMI) was 25.48 ± 3.4 kg/m^2^. The mean Deyo score was 0.63 ± 0.59 (Additional file 1: Table S1). All 19 patients in the fusion group underwent unilateral THA and spinal fusion was performed, on average, 5 years (8 months to 10 years) prior to the THA.

**Fig. 1:**
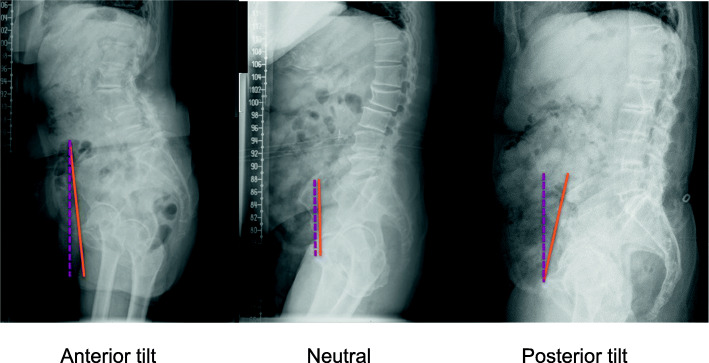
The SPT angle, which is the angle between the vertical line (purple dotted line) and the line connecting the pubic symphysis and the center of the anterior superior iliac spine (orange line) in the standing position, was divided to three categories: anterior tilt (positive value), neutral (zero degree), and posterior tilt (negative value)

All patients underwent regular THA through the Hardinge approach performed by two experienced surgeons. The standard periprosthetic infection prevention protocol and prophylactic measures for venous thromboembolism (VTE) were implemented. The patients were rehabilitated by the same rehabilitation team. We analyzed component positioning on postoperative standing anteroposterior (AP) and lateral radiographs at the 12-month follow-up visit. Inclination was defined as the angle formed between the largest diameter of the component and the inter-teardrop line. Anteversion was calculated by using the formula AV = sin-1(A/B), where A is the short diameter and B the long diameter of the component. The SPT was measured on the standing radiographs. The value was taken as positive if the pelvis was tilted anteriorly and as negative if the pelvis tilted posteriorly (Fig. 1). Additionally, the sacral inclination angle (SA), lumbar lordosis angle (LL) and pelvic coronal tilt angle (CTA) in the standing position were measured both before and 12 months after the THA. Cup inclination and anteversion were measured only after the THA in the standing AP view. The functional outcome was assessed on Harris hip function scale in terms of HSS.

### Statistical analysis

All statistical analyses were performed with STATA IC 14 (Texas, USA). Normality tests were conducted with the Kolmogorov-Smirnov test. When the factors were nonnormally distributed, a bivariate analysis among the groups was carried out with the Kruskal-Wallis test, and post hoc comparisons were also made. Categorical data were evaluated using a chi-square test or Fisher exact tests, and continuous variables were compared by the Mann-Whitney U test. Correlation was assessed by Spearman’s rank correlation coefficient. A multilinear regression analysis was done for a multivariate analysis to analyze the factors related to changes in SPT and the Harris score. *P* values < 0.05 represented significant differences and significant correlations.

## Results

### Change of SPT in two groups

**Fig. 2:**
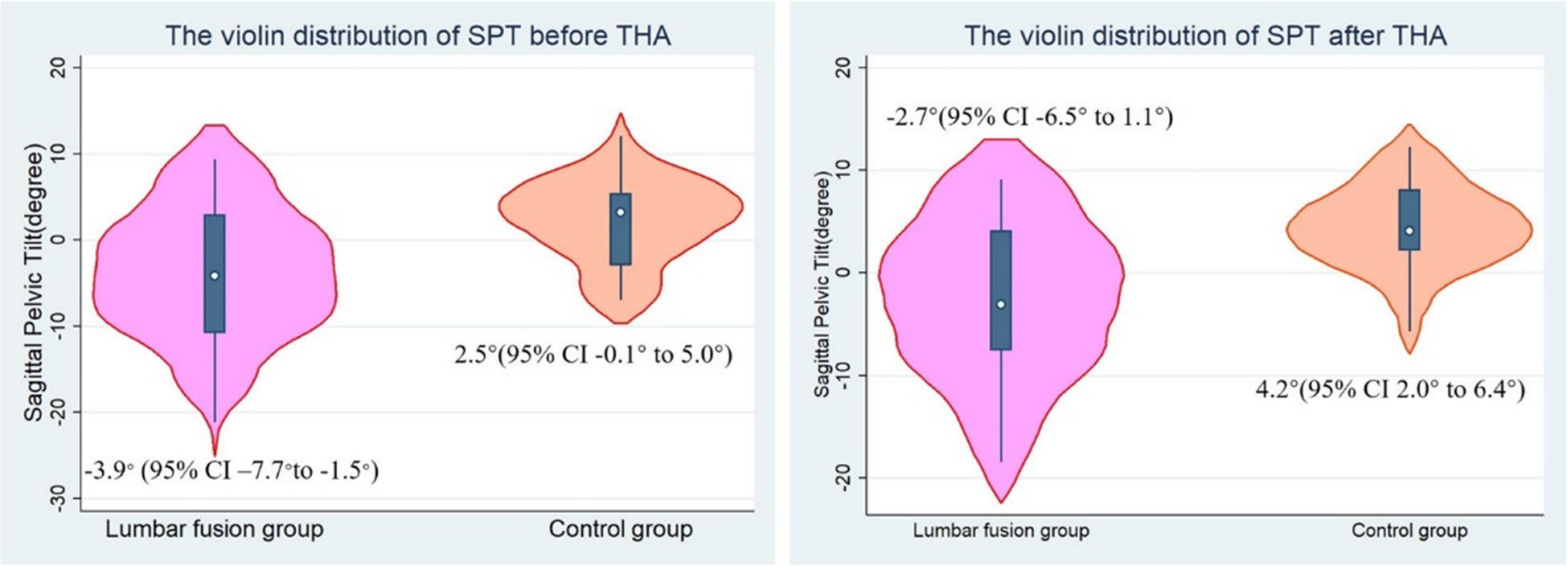
The violin plot of SPT showing that the mean SPT was significantly less both before and after THA in the lumbar fusion group, which indicates a relatively posterior SPT

**Fig. 3:**
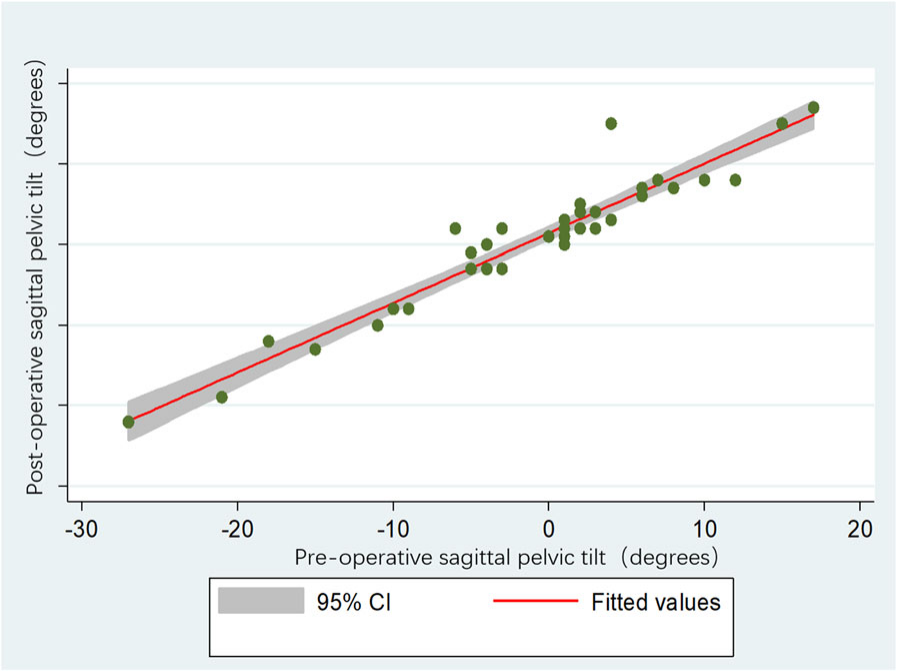
There was a positive relationship between the SPT before and after THA

The mean SPT was − 3.9° (95% CI − 7.7° to − 1.5°) in the lumbar fusion patients and 2.5° (95% CI − 0.1° to 5.0°) in the control group on the standing radiograph before THA (t = 2.27, *P* = 0.03). After THA the mean SPT was − 2.7° (95% CI − 6.5° to 1.1°) in the lumbar fusion patients and 4.2° (95% CI 2.0° to 6.4°) in the control group on the standing radiograph (t = 2.79, *P* = 0.01) (Fig. 2). The SPT before and after THA was closely and linearly correlated (coef. 0.83, *P*<0.001) (Fig. 3). The mean increase in SPT was 1.5° after THA compared to SPT before THA (t = 3.39, *P* = 0.002).

### The effects of L5S1 fusion and fusion segments on the SPT

There were 8 cases of one-segment fusion, 7 cases of two-segment fusion, 3 cases of three-segment fusion and 1 case of four-segment fusion. In total, 35 intervertebral discs were fused in 19 patients, with one L1–2 fusion, two L2–3 fusions, seven L3–4 fusions, sixteen L4–5 fusions and nine L5-S1 fusions (Additional file 1: Table S2). In the lumbar fusion patients, the mean SPT was − 3.9° (95% CI − 9.9° to 2.0°) with L5S1 fusion and − 4.0° (95% CI − 10.0° to 2.1°) without L5S1 fusion on the standing radiograph before THA (t = 0.01, *P* = 0.99). The mean SPT was − 1.2° (95% CI − 4.9° to 2.6°) with one- and two-segment fusion and − 10.0°(95% CI − 18.5° to 1.5°) with three- and four-segment fusion before THA (t = 2.60, *P* = 0.02). The SPT before and after THA was closely and linearly correlated in the lumbar fusion patients (coef. 0.97, *P*<0.001).

### Changes in other spinopelvic parameters and HHS

**Fig. 4:**
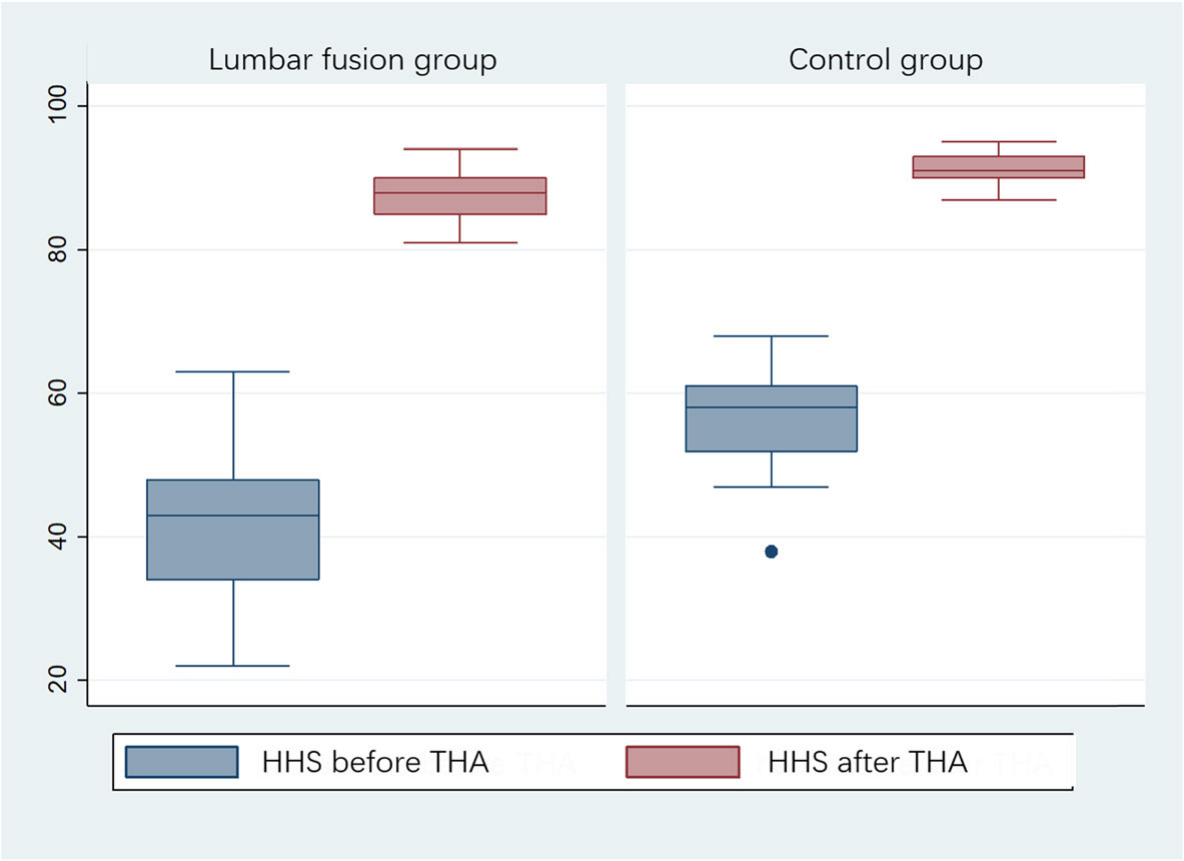
The box plot of the Harris hip score showing that the score was significantly improved after THA in both the lumbar fusion and control groups

In the study, there was no significant difference between the lumbar fusion and control groups in terms of SA, LL and CTA before THA. The HHS was higher in the control group than in the fusion group before THA (Table 1). No statistically significant difference was found between the lumbar fusion and control groups in cup inclination, cup anteversion and HHS after THA (Table 2). The Harris hip score was greatly improved in both the lumbar fusion and control groups (Fig. 4).

**Table 1:** The SA, LL and CTA in the lumbar fusion and control group were similar without a statistically significant difference. The Harris hip score before THA was higher in the control group than in the fusion group

	Mean(°)	95% CI	t value	*P*
SA				
Fusion	24.3	19.4–29.1	1.99	0.06
Control	30.1	26.2–34.0		
LL				
Fusion	31.4	31.3–40.5	1.12	0.27
Control	35.9	24.2–38.6		
CTA				
Fusion	1.4	−1.1–2.6	−0.66	0.52
Control	0.71	0.3–2.6		
Harris score				
Fusion	50.9	45.6–56.2	2.29	0.03
Control	57.8	54.2–61.2

**Table 2:** The cup inclination, cup anteversion and Harris hip score after THA in the lumbar fusion and control groups were similar without a statistically significant difference

	Mean(°)	95% CI	t value	*P*
Cup inclination				
Fusion	43.7	42.4–45.1	−1.17	0.25
Control	42.6	41.1–44.1		
Cup anteversion				
Fusion	20.3	18.7–21.8	−0.08	0.94
Control	20.2	18.9–21.5		
Harris score				
Fusion	90.1	88.0–92.3	1.20	0.24
Control	91.2	88.6–91.6

### Clinical outcomes

**Fig. 5:**
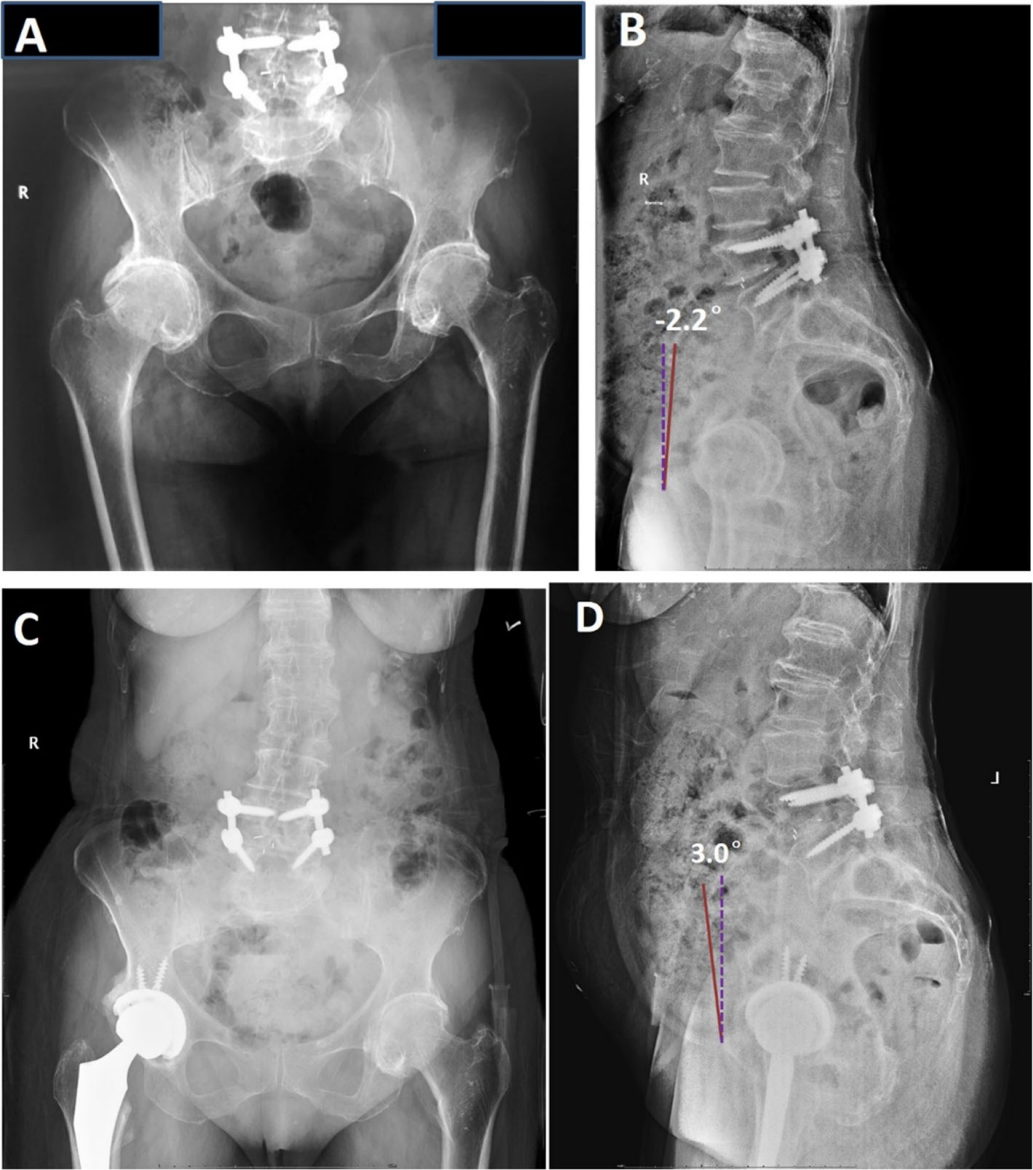
A 72-year-old female underwent total hip arthroplasty with a prior lumbar fusion. The SPT before THA (**a**, **b**) and after THA (**c**, **d**) in the standing position was −2.2 degrees (posterior SPT) and 3.0 degrees (anterior SPT), respectively

In the study, neither deaths nor serious complications occurred, such as pulmonary embolisms, periprosthetic joint infections, dislocations, fractures, nerve injuries (Fig. 5). There were 3 asymptomatic distal VTEs (1 in the control group and 2 in the fusion groups), as diagnosed by ultrasound monitoring, 2 pulmonary infections (in the fusion patients) and 1 superficial surgical site infection (in a control patient). All side effects were removed by conservative treatment.

## Discussion

The management of concurrent degenerative hip and spine pathologies entails the decision jointly made by both hip and spine surgeons. Although spine pathologies and prior spinal fusions have been cited as risk factors for THA instability, the exact relationship remains unclear [17–19]. Though sagittal imbalance in the spine has been thought to increase the risk for dislocation after THA, many questions remain unanswered regarding the unique association between sagittal imbalance and acetabular anteversion that contributes to THA instability [20].

The results of this study demonstrated that previous spinal fusion surgery increased the posterior SPT in primary THA compared with control patients, indicating that spinal fusion before THA might increase the risk for dislocation and impingement by increasing posterior SPT. SPT affects the functional orientation of acetabular components in patients undergoing THA, in that increasing posterior SPT increases functional acetabular inclination and anteversion, while decreasing SPT will result in the loss of functional inclination and anteversion [21]. SPT refers to SPT in the standing lateral position. If the SPT after THA is different from that before THA, it can be a cause of sequelae due to functional malalignment of the cup. These spinal disorders and aging itself are related to SPT changes, particularly in the standing position. Few studies mentioned the value of SPT changes, and our study might shed new light on the implication of SPT. We confirmed the SPT and spinal fusion were related and demonstrated that posterior SPT was increased by approximately 6 degrees in our cohort.

Other studies also identified spinal fusion as a significant risk factor for dislocation after primary THA, and the risk of dislocation can decrease with time, indicating that the changes in SPT at different time may be a predictor of dislocation [1, 2, 4, 15, 18, 22–24]. Gausden et al. [22] performed a meta-analysis and concluded that a history of spinal fusion was the most significant independent risk factor for dislocation within the first 6 months following THA. Furthermore, the latest study by Salib et al., found that lumbosacral spinal fusions prior to THA increased the risk of dislocation within the first 6 months and fusions involving the sacrum with multiple levels of lumbar involvement notably increased the risk of postoperative dislocation compared with a control group and individuals with other lumbar fusions. This result coincidentally confirmed the conclusion of Gausden et al. We found the posterior SPT was approximately 11 degrees more in THA patients with three- and four-segment lumbar fusion than in their counterparts with one- and two-segment fusion. The L5S1 fusion alone was not a predictor of SPT changes. The finding indicates that the long lumbar fusion may be a significant risk factor for abnormal SPT, which subsequently increases the possibility of malpositioning of cup and risk of dislocation, but L5S1 fusion is not a risk factor. The surgeons need to meticulously plan the surgery to improve the functional cup inclination and anteversion in the patients with abnormal SPT [21]. In our study, there existed no statistically significant difference in cup inclination and cup anteversion after THA in the lumbar fusion and control patients.

Whether spinal fusion exerts a positive effect or a negative effect on HHS remains debated [2, 25]. Salib et al. [2] reported that there was a small difference in the degree of improvement favoring the control group (27 points in the fusion group and 35 in the control group) but this did not reach statistical significance (p=0.07, Student’s t-test). However, Grammatopoulos et al. [25] showed otherwise: the THA-spinal arthrodesis group had lower hip functional scores compared with the THA-only group. In our study, the patients with lumbar fusion achieved HHS similar to control patients 12 months after THA despite the fact that they had different SPT and HHS before THA.

This study had several limitations. First, the sample size is too small to obtain definitively reliable results. However, to mitigate this limitation, we chose a matched case-control design. The study population was also too small to detect significant differences in individual complication rates. Second, as a retrospective study, we failed to include parameters of the sitting position. Third, several different spinal fusion levels, especially with lumbosacral fusion, may have different effects on the result [2], indicating that the result may be biased.

## Conclusion

Lumbar fusion appears to increase the posterior SPT by approximately 6 degrees in the patients undergoing THA. Lumbar fusion of more than two segments is a predictor of more posterior SPT changes, but fusion of L5S1 is not.

## Supplementary information

**Additional file 1: Table S1.** Summary of patients’ general characteristics. **Table S2.** The demographic data, lumbar fusion segments and SPT before and after THA in the patients with lumbar fusion.

## Data Availability

Please contact the authors for relevant data.

## Abbreviations

CTA: Coronal tilt angle
HHS: Harris hip score
LL: Lumber lordosis angle
SA: Sacral inclination angle
SPT: Sagittal pelvic tilt
THA: Total hip arthroplasty
VTE: Venous thromboembolism
