# Supplementary material for: How much does lumbar fusion change sagittal pelvic tilt in individuals receiving total hip arthroplasty?
Source: Arthroplasty. 2019 Nov 29;1:14. doi: 10.1186/s42836-019-0014-4 (PMC8796607; doi:10.1186/s42836-019-0014-4)
Supplement: Supplementary file 1 — Additional file 1: Table S1. Summary of patients’ general characteristics. Table S2. The demographic data, lumbar fusion segments and SPT before and after THA in the patients with lumbar fusion. [file 42836_2019_14_MOESM1_ESM.docx]

The summary of patients’ general data(table S1) and detail information of lumbar fusion segments and SPT before and after THA(table S2)

Table S1. Summary of patients’ general characteristics

| Group | Cases | Age | Gender | | Deyo score | |  |
| --- | --- | --- | --- | --- | --- | --- | --- |
|  |  |  | Male | Female |  |  | |
| Control | 19 | 62.9±6.5 | 10 | 9 | 0.57±0.60 | | |
| Fusion | 19 | 60.8±7.3 | 10 | 9 | 0.63±0.59 | | |
| P value |  | 0.10 | / | | 0.78 | | |

Table S2 The demographic data, lumbar fusion segments and SPT before and after THA in the patients with lumbar fusion.

| Case | Age（year） | Gender | Fusion segment | | | | | | | | | SPT before THA (degree) | SPT after THA (degree) |
| --- | --- | --- | --- | --- | --- | --- | --- | --- | --- | --- | --- | --- | --- |
|  |  |  | L1-2 | | L2-3 | | L3-4 | | L4-5 | | L5-S1 |  |  |
| 1 | 60 | M |  |  | | **+** | | **+** | |  | | -11.4 | -10.2 |
| 2 | 63 | F |  |  | |  | | **+** | | **+** | | -3.2 | 2.5 |
| 3 | 71 | F |  | **+** | | **+** | | **+** | | **+** | | -12.7 | -13.7 |
| 4 | 59 | F |  |  | |  | | **+** | |  | | -4.2 | -3.3 |
| 5 | 61 | M | **+** | **+** | | **+** | |  | |  | | -13.6 | -14.2 |
| 6 | 57 | M |  |  | | **+** | | **+** | | **+** | | -21.2 | -18.5 |
| 7 | 68 | F |  |  | |  | | **+** | |  | | -5.8 | -4.1 |
| 8 | 53 | M |  |  | | **+** | | **+** | |  | | -10.7 | -7.5 |
| 9 | 57 | F |  |  | |  | |  | | **+** | | 1.8 | 3.4 |
| 10 | 61 | M |  |  | | **+** | | **+** | | **+** | | -3.4 | -3.1 |
| 11 | 62 | M |  |  | |  | | **+** | | **+** | | -5.6 | -5.8 |
| 12 | 75 | F |  |  | |  | | **+** | |  | | 7.2 | 6.1 |
| 13 | 63 | M |  |  | |  | | **+** | | **+** | | 2.9 | 6.8 |
| 14 | 75 | M |  |  | |  | | **+** | |  | | 3.8 | 4.2 |
| 15 | 55 | F |  |  | |  | | **+** | |  | | 5.5 | 6.9 |
| 16 | 64 | M |  |  | | **+** | | **+** | |  | | -6.1 | -5.1 |
| 17 | 63 | M |  |  | |  | |  | | **+** | | 9.4 | 9.1 |
| 18 | 72 | F |  |  | |  | | **+** | | **+** | | -2.2 | 3.0 |
| 19 | 56 | F |  |  | |  | | **+** | | **+** | | -5 | -2.9 |
